# The Effect of Sleep Loss on Retrospective Metacognitive Judgements Across Five Cognitive Tests

**DOI:** 10.1111/jsr.70141

**Published:** 2025-08-14

**Authors:** Tina Sundelin, Andreas Jemstedt, Alvin Gavel, Bennett L. Schwartz, John Axelsson

**Affiliations:** ^1^ Department of Psychology Stockholm University Stockholm Sweden; ^2^ Department of Clinical Neuroscience Karolinska Institutet Stockholm Sweden; ^3^ Department of Education Stockholm University Stockholm Sweden; ^4^ Independent Researcher Stockholm Sweden; ^5^ Department of Psychology Florida International University Miami Florida USA

**Keywords:** cognitive performance, metacognition, metacognitive accuracy, sleep deprivation, sleepiness

## Abstract

Sleep loss impairs many cognitive functions, ranging from simple attention to working memory. This study explores the extent to which people are aware of such impairments, their metacognitive accuracy, across different cognitive tests. Healthy participants (*N* = 182) were randomised to one night of total sleep deprivation or three nights of sufficient sleep. The next day they performed several cognitive tests, measuring simple attention, cognitive throughput, working memory, episodic memory and executive processing (using a Stroop task). After each test, participants rated how well they thought they performed. We operationalised metacognitive accuracy as the ability to correctly identify whether one performed above or below the median. We then used Bayesian methods to estimate the difference in this ability between the well‐rested and sleep‐deprived groups. The probability was 55% in the sleep‐deprived group, and 59% in the rested group, suggesting some decrease in performance awareness during sleep loss. However, the probability that this difference in judgements is practically significant (i.e., exceeding 10 percentage points) is below 1%. Cognitive ability generally declines during sleep deprivation, and this was at least somewhat reflected in a decrease in how people rated their performance. The question remains whether and how people compensate for any sleep‐loss induced cognitive impairments.

## 1 Introduction

Sleep loss has a negative impact on a range of cognitive abilities, from simple attention to working memory and executive functioning (Lim and Dinges 2010; Lowe et al. 2017). Although performance is generally impaired after insufficient sleep, there is reason to believe that one would also expect to perform worse. Sleep loss likely increases the feeling of effort associated with performing tasks (Engle‐Friedman 2014), which could influence people's thoughts about their performance (Alter and Oppenheimer 2009). In addition, people may also hold personal beliefs that sleep loss lowers their ability to perform (Mueller and Dunlosky 2017). However, the extent to which one can gauge the effect of sleep loss on performance has not been well studied. Considering that insufficient sleep and severe sleepiness are common in many safety‐critical occupations, such as for shift‐working medical doctors, a better understanding of how metacognitive abilities are affected can help prevent negative outcomes.

## 2 Metacognition and Sleep

Metacognition is a person's awareness and knowledge of their cognition and the resulting effects of that awareness and knowledge on their behaviour. Metacognition involves monitoring one's ongoing cognitive activities (i.e., metacognitive monitoring), such as learning, retrieval or problem‐solving, and then regulating one's thoughts, emotions, motivation, environment and behaviour to achieve one's goals (i.e., metacognitive control) (Nelson and Narens 1990; Pintrich 2000). Metacognition includes one's knowledge about one's cognition (i.e., metacognitive knowledge), such as one's strengths and weaknesses in relation to different tasks and strategies for thinking and problem‐solving, such as what learning methods work best for that individual (Karabenick et al. 2021; Pintrich 2002). Such prior knowledge can, for example, influence the monitoring and use of one's cognitive effort (Mueller and Dunlosky 2017). The finding that the brain/cognition system can maintain performance during sleep loss by recruiting additional brain areas (Drummond et al. 2000) supports the notion that we can engage planning and effort components to mitigate sleep‐loss‐induced cognitive impairments.

The literature on the effects of sleep loss on metacognitive judgements is limited, and the existing studies report conflicting findings (for reviews, see Boardman et al. 2021; Jackson et al. 2018). Although participants are typically more conservative in rating their performance when sleep‐deprived (Dorrian et al. 2000), some studies find that sleep deprivation makes participants more overconfident about their performance, whereas others find underconfidence, higher accuracy, or no effects at all. Even in studies with long, within‐subject sleep deprivation protocols, in which participants stayed awake for 40–66 h, the effects were inconsistent (Aidman et al. 2019; Baranski et al. 2002, 1994; Boardman et al. 2018).

One possible explanation for these differences is that they stem from the wide variation in metacognitive measures, sleep protocols, and cognitive tasks used in previous research. However, even within one type of metacognitive judgement, namely retrospective confidence judgements about overall performance on a test, results have varied (Baranski 2007; Blagrove and Akehurst 2000; Boardman et al. 2018; Kilpeläinen et al. 2010). One study using the same error monitoring task for two different types of sleep manipulations (one night of total sleep deprivation vs. three nights of sleep restriction) found that restricted participants were less aware of errors they made, but there was no effect of total sleep deprivation relative to a full night's sleep (Boardman et al. 2024). Some of the variation between studies is likely because of the small number of participants (Aidman et al. 2019; Jackson et al. 2018), which makes the conclusions more susceptible to random error. In addition, there has been little focus on the potential mechanisms behind a potential relationship between metacognition and sleep loss, such as how feeling sleepy affects metacognition independent of objective sleep loss.

## 3 Underlying Mechanisms

To understand the conflicting findings, there are several potential mechanisms to consider, factors through which sleep loss could, theoretically, affect metacognition. First, with respect to metacognitive knowledge, people sometimes have strong beliefs about the effect of sleep loss on cognition, which may affect metacognitive judgements. Indeed, many expect that not getting enough sleep affects their cognition (Krizan and Hisler 2021). Undoubtedly, there are also some people who do not believe sleep loss affects their cognitive abilities. Thus, one's beliefs about how the personal effects of sleep loss need not be accurate, and both these beliefs and their accuracy likely vary across individuals. Thus, it is possible that many overestimate or underestimate the effect of sleep loss. Note that these estimations are not directly the result of sleep loss. Rather, they come from beliefs about the result of sleep loss.

Insufficient sleep may also affect metacognition directly, through altering the processes that lead to metacognitive judgements. For example, processing fluency, the experience of mental ease or effort when processing information (Alter and Oppenheimer 2009; Undorf 2020) has repeatedly been shown to be an important factor in many metacognitive judgements (Jemstedt et al. 2018; Schwartz and Jemstedt 2021; Undorf 2020). In general, higher processing fluency (less effort) is associated with more optimistic or higher judgements, and lower processing fluency is associated with more pessimistic or lower judgements, regardless of how such fluency affects the underlying cognition (Jemstedt et al. 2018). As sleep deprivation generally makes tasks feel more effortful and less motivating (Axelsson et al. 2020; Engle‐Friedman 2014), this could affect processing fluency, resulting in more pessimistic metacognitive judgements. Accordingly, more alertness is related to higher self‐ratings of performance (Dorrian et al. 2000, 2003). Thus, it is highly likely that sleep deprivation will cause lower metacognitive judgements when processing fluency is a factor, as a result of feeling sleepy. Sleep‐deprived individuals may thus experience their performance on a task as worse because it required more cognitive effort than after a good night's sleep.

People rely on a host of heuristic information to inform metacognitive judgements, and people's reliance on different information changes according to context (Koriat 1997; Metcalfe 1993; Undorf et al. 2018). As such, sleep deprivation could also affect metacognitive judgements by influencing what information people rely on and under what circumstances, as well as how they integrate that information when making their judgements. When people monitor their cognition, they rely on heuristic cues at the moment of judgement and use these heuristics to translate those experiences into judgements (Koriat 1997). For example, they might note that they are able to visualise all the words they are trying to memorise and, therefore, judge that they have successfully stored them in memory, or they might judge that they will not remember a word in an hour because it took a while to recall it from memory now. Because people vary in which and how many cues they rely on (Undorf et al. 2018), it is possible that sleep loss affects metacognitive judgements either by limiting the number of cues people incorporate into their judgements or by making it more likely that one cue will gain influence over another. For example, sleep‐deprived individuals may be more likely to rely on their experience of low processing speed or greater variance in processing speed than well‐rested individuals who generally experience higher processing speed and less variance. Sleep deprivation could also affect how different cues are weighted when translated into judgements, as priorities in motivation and effort shift when sleepy (Axelsson et al. 2020; Massar et al. 2019).

## 4 Metacognitive Accuracy

When people monitor their cognition to predict how they will perform on an upcoming task, assess how a task is going, or evaluate how they performed on a recent task, their judgements vary in the accuracy with which they predict performance (Jin et al. 2022). If a student just studied for tomorrow's exam and judged that they would do well on it but then performed poorly, this would be an example of an inaccurate judgement. However, if they had expected to perform poorly, the accuracy would have been good. Thus, the accuracy of metacognition is its mapping to the actual performance rather than the positivity or negativity of the judgement or the absolute level of performance itself. Whether or not a metacognitive judgement is accurate depends on the validity of the heuristic cues the judgement relies on and the quality of the heuristics used to translate the underlying metacognition into judgements (Koriat 1997). For example, if a student notes that they are tired and therefore judges that they will perform poorly on a test but, in fact, the sleep loss had no substantial effect on their performance, this will result in lower judgement accuracy. Similarly, if an individual relies on processing fluency as a cue and judges that, because it was mentally straining to memorise a list of words, they did not perform well on a memory test, this will result in lower accuracy if processing fluency was not a valid predictor.

In metacognitive research, there is a distinction between two types of accuracy: absolute and relative accuracy, also called calibration and resolution, respectively (Dunlosky and Metcalfe 2009). Absolute accuracy, or calibration, is the match between the overall performance on a task and the judged performance. If you judge that you will remember 8 out of 10 items on a memory test but only remember 5 items, you would have been overconfident with fairly low calibration/absolute accuracy. Meanwhile, relative accuracy, or resolution, requires judgement on an item‐by‐item level. For example, if you judge that it is more likely that you will remember items A, B and C than items D, F and G, and then you actually remember items A, D, F and G on the test, your resolution/relative accuracy is fairly poor. Although sleep loss may affect both types of accuracy, the focus of the present study is on calibration/absolute accuracy.

In addition, there is a contrast between global and local, item‐by‐item, confidence. Global confidence means that participants provide a task‐wide estimate of how they performed on that task, which contrasts with local, or item‐by‐item metacognitive judgements, in which people rate their performance on each item separately (Fleming 2024). Research shows that the basis for local and global metacognitive judgements may be quite different, with global judgements being more based on an individual's beliefs than local judgements are (Händel et al. 2020). In addition, global judgements tend to be more overconfident than item‐by‐item metacognitive judgements (Kröner and Biermann 2007). We suspect that both global and local judgements could be sensitive to differences in amount of sleep, but for different reasons. Global judgements may be more affected by one's belief about how sleep affects performance, whereas local judgements may be more sensitive to the effects of sleep loss on mental effort, making processing of information less fluent. Although sleep loss may affect both global and local judgements, the focus here is on global judgements.

## 5 The Present Study

In the present study, participants who have either been sleep‐deprived or had a full night of sleep completed a battery of cognitive tests several times the next day. Each time they completed a test, they judged how they performed overall on that test. They also rated their sleepiness along with each test, as a potential underlying mechanism between sleep loss and metacognitive ratings and accuracy.

Using this data, we investigated the following research questions:

1. To what degree does sleep loss and sleepiness influence the magnitude of retrospective metacognitive judgements?
2. To what degree does sleep loss and sleepiness affect the absolute accuracy of retrospective metacognitive judgements?
3. Is the influence of sleep loss and sleepiness on retrospective metacognitive judgements and their accuracy similar across our cognitive tasks, or does it vary?

## 6 Method

### 6.1 Participants

Healthy participants (*N* = 182, age range 18–45, 103 women) took part in this between‐subject sleep‐deprivation study. They were screened for general physical and mental health problems, as well as sleep problems. Exclusion criteria also included a sleep need outside of 7–9 h, being a smoker or drinker, and having trouble abstaining from caffeine. A full list of screening criteria can be found in a previously published study (Holding et al. 2019). Participants were divided into a sleep‐deprivation group (*N* = 91, average age = 25.4, SD = 6.2, 57% women) and a normal‐sleep group (*N* = 91, average age = 25.3, SD = 6.8, 56% women). The study was approved by the regional ethical review board in Stockholm (2014/1766‐32).

### 6.2 Measures

*Cognitive performance* was measured using the Karolinska WakeApp (KWA; Holding et al. 2021), with five brief tests assessing simple attention, cognitive throughput, working memory, episodic memory, and executive processing; see below for a closer description of each. All tests were performed on a smartphone, either the participants' own or one provided by the researchers if participants did not have one.

The simple attention task used a setup similar to a psychomotor vigilance task (e.g., Roach et al. 2006). Participants were instructed to keep their attention on the screen, reacting as quickly as possible by pushing a ‘button’ on the screen as soon as the letter ‘p’ showed up. The p was always in the middle of the screen. The performance measure is the average reaction time between the p being displayed and the participant touching the button on the screen. The test lasted 2 min.

Cognitive throughput indicates processing speed, based on the number of correct responses on a task within a given time (e.g., Brieva et al. 2021). The task used here was an arithmetic test, in which participants were instructed to, ‘as quickly as possible and as correctly as possible’, provide the answer to an addition of two numbers with two digits each between 11 and 99, for example ‘47 + 36’. The performance measure is the number of correct responses given in 2 min.

Working memory was assessed using a grid task, specifically indicating spatial working memory (Klingberg et al. 2002). Participants were presented with a 4 × 4 grid, in which seven of the squares flashed briefly in red, in a random order. Following this, one of the squares would turn red while displaying a number, and participants were instructed to answer whether that number correctly indicated the order in which that square had flashed red. In other words, if the red square showed the number 4, participants responded yes or no to whether that square had been the fourth out of the seven to flash red. Participants did 10 rounds of this, and the performance measure is the percent correct responses out of the 10. Note that because of a programming error, seven out of the 613 sessions had 12 rounds rather than 10. As the results are in percent correct, these sessions were included without further focus.

For episodic memory, participants were instructed to memorise a list of 12 words, which was presented on the screen for 12 s, followed by a fixation cross for 5 s. They were then presented with the 12 words mixed with 12 new words and asked to indicate for all 24 words whether or not they had been on the list. They were then shown the original list for another 12 s, followed by a fixation cross for 5 s. Following this, they were presented with the original 12 words mixed with 12 new words and asked to indicate which ones they had seen before (often know as an old/new recognition test). The performance measure is the number of correct responses out of the 48 responses.

Executive processing was assessed using the Stroop test (Stroop 1935), in which a word indicating a colour (e.g., ‘green’) would appear on the screen. Participants were instructed to ‘as quickly as possible and as correctly as possible’, indicate the colour of the font of the word, ignoring the meaning of the word. The word could either be congruent with the font colour (e.g., the word ‘green’ written in green font) or incongruent with the font colour (e.g., the word ‘green’ written in red font). Participants responded by pressing the ‘button’ on the screen representing the correct colour out of four possible (red, yellow, green, blue). These were indicated by words rather than colours. The test ran for 2 min, including both congruent and incongruent trials. As accuracy is usually quite high (and was found to be consistently above 95% in this sample in a previous report; Holding et al. 2021), the performance measure is the median reaction time across all trials.

*Sleepiness* was measured with a slightly modified version of the Karolinska Sleepiness Scale (Åkerstedt and Gillberg 1990), to suit a mobile phone device, ‘How sleepy are you right now?’, using a 9‐point scale ranging from 1—Extremely awake to 9—Extremely sleepy.

*The metacognitive judgement perceived performance* was measured with the question ‘How did you perform on the test?’, using a 9‐point scale ranging from 1—Very Poorly to 9—Very well.

### 6.3 Procedure

Following recruitment and screening, participants came to the sleep lab to give informed consent and practise the tests. Participants were given an activity monitor (GeneActiv Sleep, Activinsights Limited, Kimbolton, UK) to wear on their non‐dominant wrist and instructed to spend 8–9 h in bed/night for three consecutive nights. They were to turn off the light at 23:00 h ± 60 min and get up at 07:00 h ± 60 min. On the afternoon of the day after the third night, they were informed of their sleep group for the fourth night and either instructed to spend one more night at home according to this schedule or come to the sleep lab at 22:00 that evening and stay awake throughout the night. All participants did an additional round of the cognitive tests that evening. The sleep‐deprived participants were monitored throughout the night in the lab, ensuring they did not fall asleep. They engaged in activities such as studying, reading, watching movies/TV‐shows, etc.

During the day after the experimental night (i.e., the fourth night), participants did all the cognitive tests three times: in the morning (around 08:00), after lunch (around 12:30), and in the afternoon (around 16:30). Following each test, they indicated how sleepy they felt and their perceived performance, resulting in five responses at each time point.

### 6.4 Analysis

For our analyses, we operationalised metacognitive ability as the ability of participants to correctly identify whether they perform better or worse on average, compared to other people. We then estimated whether that ability differed between the well‐rested and sleep‐deprived group to an extent likely to be of scientific and practical interest. This analysis method was intended to use the data in the most conservative way, without too many statistical assumptions.

There are two relevant unknowns. First, as participants rated their performance on a scale from 1 to 9, we do not know what aspect of their performance they had in mind (e.g., speed, accuracy, errors etc.). Therefore, we cannot directly test how well their ratings reflect our metrics of their performance. For example, if a participant has answered 80% of questions correctly on the working memory test and then given themselves the rating ‘ 7’ we cannot compare the performance and rating to say how accurate that rating is. As far as we know, they might have correctly estimated that they got 80% right and happen to feel that this performance merits a rating of 7—or they might be overconfident, believing that they got 95% right, but feel that the test was easy enough that their performance still only deserves a 7. In the former case, the participant has good metacognitive calibration and in the second, worse calibration, but the numbers reported to us will be the same.

Second, we do not know whether the participants rated their performance in ‘absolute’ terms or if they took their sleep status into account at the time of judgement, that is, more ‘relative’. In other words, a sleep‐deprived individual might think of their performance as ‘this was terrible compared to my usual performance’ or ‘I did pretty well, considering my lack of sleep’. These would likely result in very different ratings.

To allow for these unknowns, we made one assumption for the statistical analyses. We assumed that given perfectly accurate metacognition, the ratings would be an increasing function of performance, at least within each sleep group. This implies that for perfect metacognition, participants' ratings would be ordered the same as their actual performance, with the best performer rating themselves highest, the second‐best rating themselves second best, and so on—even if we do not know which specific ratings they would give themselves.

To simplify the analyses, we did not look at the entire ordering. Instead, we simply assumed that with perfect metacognition, participants who perform below the median would rate themselves below the median, and participants who perform on or above the median would rate themselves on or above the median. Our metric for metacognitive performance in the sleep‐deprived and well‐rested group is therefore the probabilities that participants in the sleep‐deprived (*P* ^depr^) and well‐rested group (*P* ^rest^), respectively, correctly rated themselves relative to the median. A person who does poorly on the tests should rate themselves below the median, whereas a top performer should rate themselves above the median.

This leads to the question of which median to compare participants to. As mentioned above, we do not know whether participants rated their performance on an absolute or relative scale. If we assume that participants are rating their performance on an absolute scale, it would be appropriate to compare participants to the median *across* both the sleep‐deprived and well‐rested group. If we assume that participants are rating their performance on a relative scale, taking into account their sleep status, we should instead compare them to the median *within* each group. As we do not know, we made both comparisons to ensure that they do not lead to different conclusions.

Finally, as we expected that the effects of sleep‐deprivation on metacognition are similar enough between the tests that we aggregated them into a single analysis. However, as an exploratory analysis, we also show whether our conclusions would differ if they were based on each type of test.

Following Goodman et al. (2019), we did not attempt to falsify the null hypothesis that 𝑃^𝑑𝑒𝑝𝑟^ = 𝑃^𝑟𝑒𝑠𝑡^, because, as they are continuous parameters, it is a priori given that they must differ to some extent. With a large enough sample, a statistical difference would be found eventually. Instead, we estimated the probability that the difference 𝐷 between them exceeds a threshold for a *practically significant difference*, which we have—somewhat arbitrarily—set at 10%. *Practically significant differences mean that there are differences among conditions at a level of psychological interest and applied value*. In addition, we made robustness analyses for the thresholds of 5% and 1%. We discuss the mathematical details of our analysis in more detail in Appendix S1.

For the research question of the degree to which sleep loss and sleepiness influence the magnitude of retrospective metacognitive judgements, we made an exploratory analysis of how prone participants in either group were to give themselves ratings on or above the median across both groups. The formal details of this analysis are almost identical to the main analysis. We estimate the probabilities ($P_{\mathrm{rat}}^{\text{depr}}$) and ($P_{\mathrm{rat}}^{\text{rest}}$) that participants in each group give themselves a rating on or above the median, then convolve the resulting probability distributions for the probability distribution over the difference ($D_{\mathrm{rat}}$) between these. Again, we treat a difference above 10% as practically significant.

We also explored the effects of participants' self‐rated sleepiness. We then split the data into groups of above‐ and below‐median self‐reported sleepiness, as sleepiness is a robust effect of sleep deprivation (see, e.g., Axelsson et al. 2020 for sleepiness distribution across a subset of the current sample), and a potential mechanism. We discuss the robustness of these findings more in Appendix S1.

## 7 Results

### 7.1 Objective and Self‐Rated Performance

Performance on the different tests across the day has been reported previously (Holding et al. 2021). The full results and analysis code can be found on https://github.com/AlvinGavel/Too_Tired_to_Know.

Table 1 shows the medians for actual and self‐rated performance, and self‐reported sleepiness, for each group and test type, for each session. There is a tendency for actual performance to be lower in the sleep‐deprived group (worse median performance on all tasks except working memory, but see Holding et al. 2021 for detailed analyses), and there is a clear difference in self‐reported performance.

**TABLE 1:** Performance, performance judgements, accuracy, and sleepiness across groups, cognitive tests, and session number (1 = morning, 2 = midday, 3 = afternoon).

Session	Cognitive throughput		Episodic memory		Working memory		Executive processing		Simple attention	
	Sleep‐deprived	Rested	Sleep‐deprived	Rested	Sleep‐deprived	Rested	Sleep‐deprived	Rested	Sleep‐deprived	Rested
Median actual performance										
1	8.0 $\frac{\text{correct}}{\min}$	8.8 $\frac{\text{correct}}{\min}$	89.6%	93.8%	80%	80%	1191 ms	1133 ms	503 ms	474 ms
2	7.8 $\frac{\text{correct}}{\min}$	9.4 $\frac{\text{correct}}{\min}$	91.7%	93.8%	80%	80%	1103 ms	1061 ms	504 ms	470 ms
3	8.3 $\frac{\text{correct}}{\min}$	10.1 $\frac{\text{correct}}{\min}$	91.7%	93.8%	80%	80%	1112 ms	1060 ms	536 ms	491 ms
Median self‐reported performance										
1	5	5	5	6	4	5	6	6.5	5	6
2	6	6	6	6	5	5	6	7	6	7
3	5	6	5	7	4	5	5	6	5	6
Median self‐reported sleepiness										
1	7	5	7	4	8	4	8	5	7	5
2	7	3	7	3	7	3	7	3	7	3
3	8	4	8	4	8	5	8	4	8	4

*Note*: Cognitive throughput (number of correct calculations), episodic memory (% words recalled correctly), working memory (spatial accuracy %), executive processing (reaction time on Stroop), simple attention (reaction time).

**FIGURE 1:**
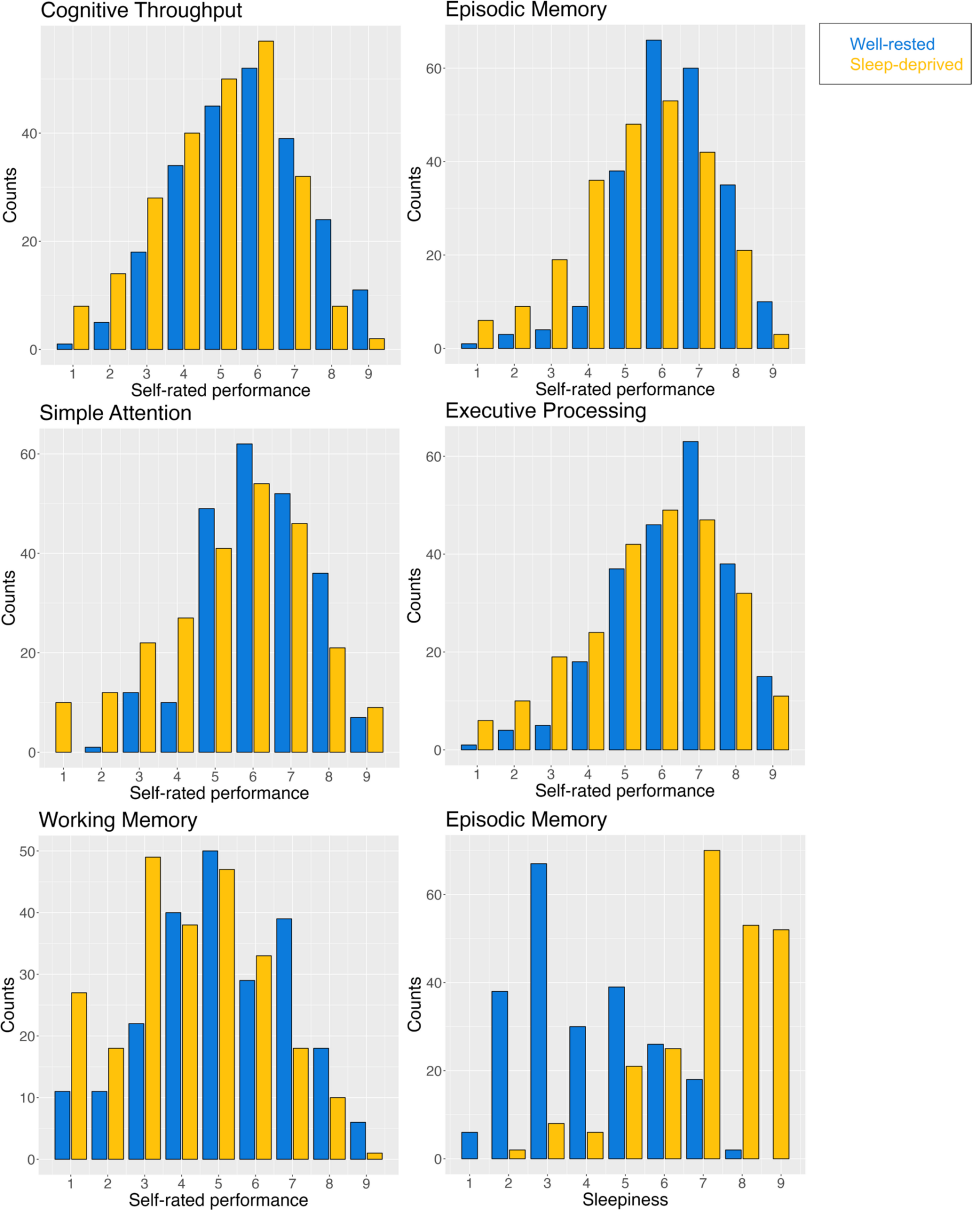
Distribution of responses on the self‐rated performance scales for each test and distribution of subjective sleepiness rated after the episodic memory test. Sleepiness is only shown for the episodic memory test as the distributions were virtually identical across tests, illustrating the large differences in subjective sleepiness between conditions.

In aggregate, 68% of ratings from the well‐rested group were on or above the median rating, while 51% of ratings from the sleep‐deprived group were. The credible interval of *D*_rat_ covers the interval −0.20 to −0.14, and the probability of a practically significant difference is 99.96%. See Table 2 for the distribution for each separate test, and Figure 1 for the distribution of metacognitive ratings and sleepiness across the scale for the two groups.

**TABLE 2:** Fraction of participants rating themselves on or above the median (across groups) depending on sleep group and cognitive test.

	On or above the median		Credible interval of *D*_rat_	Probability of practically significant difference (%)
	Well‐rested (%)	Sleep‐deprived (%)		
Cognitive throughput	68	56	−0.20 to 0.05	70
Episodic memory	76	50	−0.32 to −0.18	99.97
Working memory	63	45	−0.25 to −0.10	95
Executive processing	65	50	−0.22 to −0.07	83
Simple attention	69	54	−0.22 to 0.07	86

### 7.2 Metacognitive Accuracy

**FIGURE 2:**
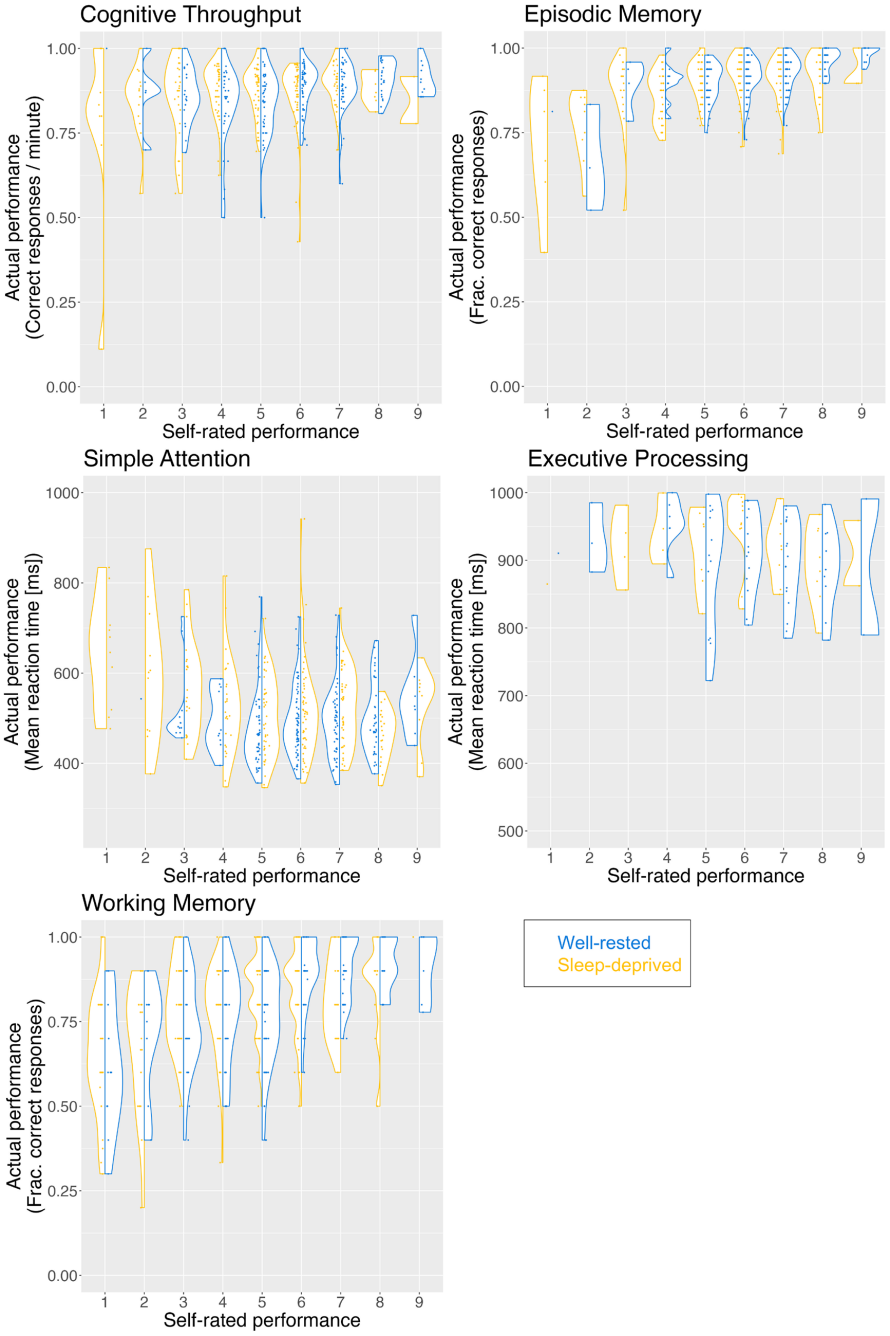
Scatterplots over self‐rated and actual performance for each of the tests. As self‐rated performance has discrete values, random jitter has been added to prevent points from overlapping. The included cognitive domains and tests were cognitive throughput (arithmetic ability), episodic memory (word memory), simple attention (go test), executive processing (Stroop), and working memory (spatial). Self‐rated performance was rated on a scale from 1 (very poorly) to 9 (very well) after each test carried out at four time points in all individuals. Frac., fraction.

**FIGURE 3:**
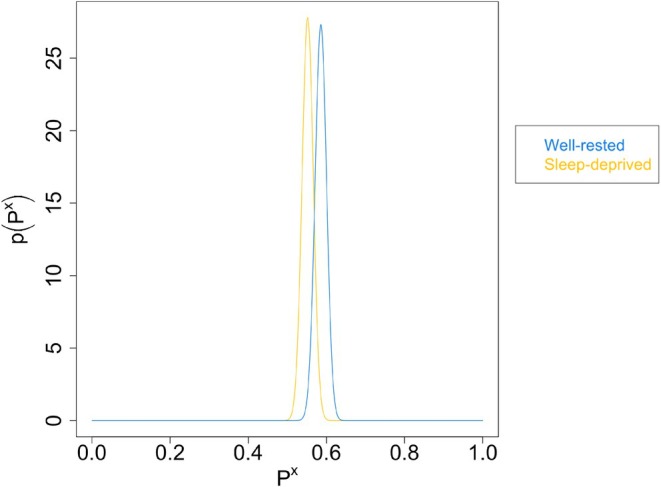
Probability distributions over the probabilities $P^{\text{depr}}$ and $P^{\text{rest}}$.

**FIGURE 4:**
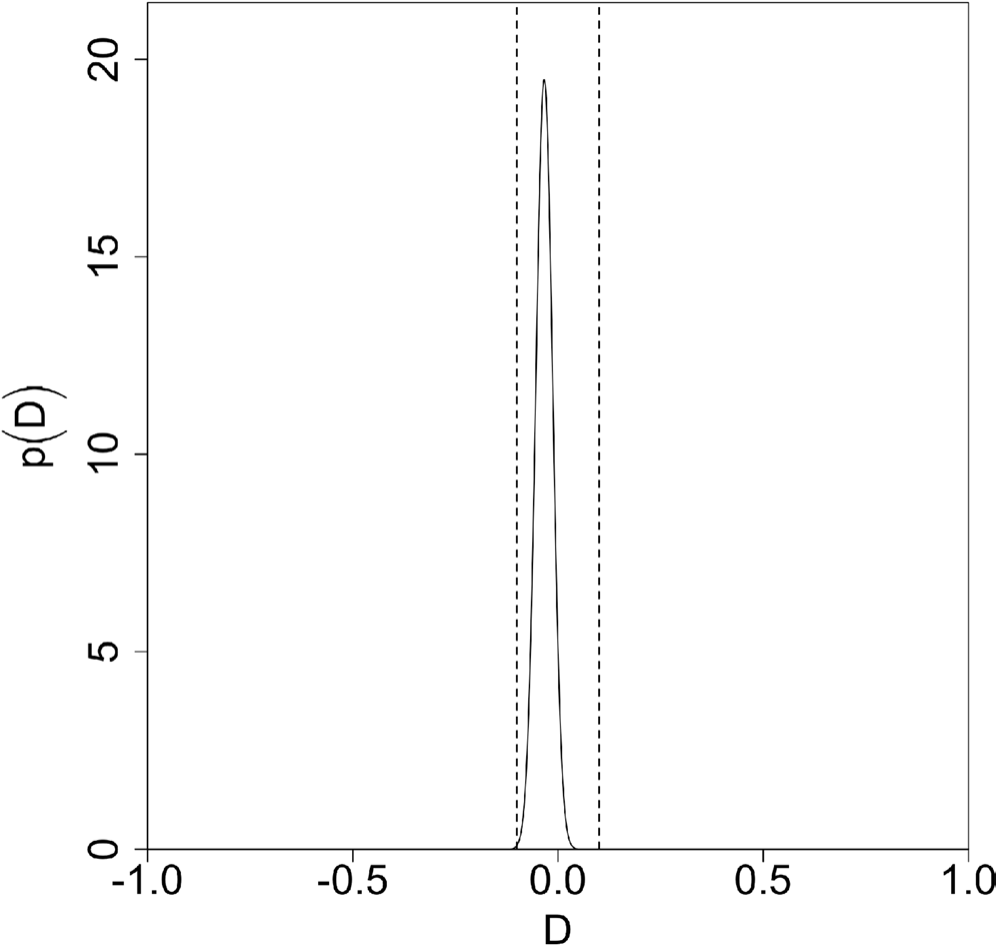
Probability distribution over the difference $D=P^{\text{depr}}-P^{\text{rest}}$. Dashed lines denote our bounds for practical significance. Only 0.06% of the probability mass is outside of the bounds, corresponding to the probability that there is a practically significant difference.

Figure 2 shows split violin plots of self‐rated performance and actual performance, with scatter for each participant. Ratings were compared to medians across both groups (i.e., including all participants) as well as within each group (i.e., each sleep condition separately). In the sleep‐deprived group, 55.21% rated themselves correctly with respect to the median (i.e., either correctly above or correctly below) across all participants, and 55.05% rated themselves correctly with respect to the median within their (sleep‐deprived) group. For the well‐rested group, the corresponding numbers were 58.58% and 59.19%, respectively. Figure 3 shows the probability distribution over $P^{\text{depr}}$ and $P^{\text{rest}}$ based on the across‐groups median that we derived from this—see Appendix S1 for details on procedure. The corresponding plot for the within‐groups medians looks almost identical. The credible interval—defined as the integral containing the central 90% of the probability mass—covers [0.53, 0.58] for $P^{\text{depr}}$ and [0.56, 0.61] for the $P^{\text{rest}}$. Convolving the two probability distributions gives the probability distribution over D, shown in Figure 4. Based on the median across groups, the credible interval for D is [−0.07, 0.00], and the posterior probability that the difference is practically significant is 0.06%; for the medians within groups, the probability is 0.2%. In other words, there is almost certainly no practically significant difference between the two groups, regardless of which median is used. See Table 3 for the probability of a practically significant effect and credibility intervals depending on which median was used.

**TABLE 3:** Probability of practically significant difference (10% or larger) depending on grouping (sleep condition or reported sleepiness) and which median was used (across or within groups); credibility interval for the probability of correct ratings in the sleep‐deprived ($P^{\text{depr}}$) and well‐rested groups ($P^{\text{rest}}$); and the difference between the groups (D). $D=P^{\text{depr}}-P^{\text{rest}}$. For the sleepiness analyses, $P^{\text{depr}}$ reflects above or on median sleepiness and $P^{\text{rest}}$ below median sleepiness.

	Probability of practically significant difference (%)	Credible interval for $P^{\text{depr}}$	Credible interval for $P^{\text{rest}}$	Credible interval for D
Assigned sleep group				
Median across groups	0.06	0.53–0.58	0.56–0.61	−0.07 to 0.00
Median within groups	0.2	0.53–0.57	0.57–0.62	−0.07 to −0.01
Sleepiness ratings				
Median across groups	> 0.00	0.54–0.59	0.55–0.60	−0.05 to 0.02
Median within groups	> 0.00	0.55–0.60	0.57–0.62	−0.05 to 0.02

### 7.3 Varying Significance Levels

We also examined what conclusions would have been drawn if we had chosen a narrower boundary for practically significant difference. We do not look at wider bounds because the probability of a significant difference at the chosen level (10%) is almost zero. We avoid clutter by showing only the results for the across‐groups median. Given a bound of 5% for a practically significant difference, the probability rises to 21%, and for 1% it rises to 89%. That the probability rises sharply for narrow bounds is expected because the limited information in the sample means that the prior comes to dominate. We discuss this more in Appendix S1.

### 7.4 Individual Tests

As another exploratory analysis, we also looked at the conclusions that would have been drawn regarding metacognitive accuracy based on the individual tests. To avoid cluttering the text with almost‐identical percentages, we split based on sleep group and compared to the across‐groups median. Based on only the cognitive throughput test, we would have found a 3.5% probability of significant difference; for the episodic memory test a probability of 16%; for the simple attention test 5.6%; for the executive processing test 3.4%; for the working memory test 39%. This means that even for the test for which the probability is the largest, that is working memory, there is still a 61% probability that there is no practically significant difference. See Table 4 for credibility intervals for the individual tests.

**TABLE 4:** Probability of practically significant difference (10% or larger) that would have been estimated using only the data from each individual cognitive task; corresponding credibility interval for the probability of correct ratings in the sleep‐deprived ($P^{\text{depr}}$) and well‐rested groups ($P^{\text{rest}}$); and the difference between the groups (D).

	Probability of practically significant difference (%)	Credible interval for $P^{\text{depr}}$	Credible interval for $P^{\text{rest}}$	Credible intervalfor D
Cognitive throughput	3.5	0.52–0.63	0.51–0.62	−0.06 to 0.09
Episodic memory	16	0.57–0.67	0.63–0.73	−0.13 to 0.02
Executive processing	3.4	0.45–0.56	0.46–0.57	−0.09 to 0.06
Simple attention	5.6	0.41–0.52	0.44–0.55	−0.10 to 0.05
Working memory	39	0.54–0.64	0.62–0.73	−0.16 to −0.01

*Note*: $D=P^{\text{depr}}-P^{\text{rest}}$. All values are based on the median across groups.

### 7.5 Sleepiness

If the splitting had been based on self‐reported sleepiness instead of assigned sleep group, 56.3% or 57.5% of ratings for the sleep‐deprived were accurate—again depending on whether the comparison is made to the across‐groups or within‐groups median. The corresponding numbers for the well‐rested group are 57.5% and 59.1%, respectively. In either case, the probability of a practically significant difference between groups is below 0.01%. Table 5 shows the medians for actual and self‐rated performance for those higher and lower in sleepiness, for each test type, for each session. See Table 3 for the probability of a practically significant effect and credibility intervals depending on whether splitting was based on reported sleepiness or assigned sleep group, and which median was used.

**TABLE 5:** Performance, performance judgements, accuracy, and sleepiness divided by high and low sleepiness using the entire sample, independently of sleep group, separated by session number (1 = morning, 2 = midday, 3 = afternoon) and cognitive test.

Session	Cognitive throughput		Episodic memory		Working memory		Executive processing		Simple attention	
	More sleepy	More alert	More sleepy	More alert	More sleepy	More alert	More sleepy	More alert	More sleepy	More alert
Median actual performance										
1	7.8 $\frac{\text{correct}}{\min}$	9.6 $\frac{\text{correct}}{\min}$	89.6%	93.8%	80%	82%	1171 ms	1150 ms	501 ms	473 ms
2	7.5 $\frac{\text{correct}}{\min}$	9.6 $\frac{\text{correct}}{\min}$	91.7%	91.7%	80%	80%	1106 ms	1061 ms	515 ms	470 ms
3	8.3 $\frac{\text{correct}}{\min}$	10.1 $\frac{\text{correct}}{\min}$	91.7%	93.8%	80%	82%	1103 ms	1062 ms	534 ms	490 ms
Median self‐reported performance										
1	5	6	6	7	4	5	6	7	5	6
2	5	6	6	6	4	5	6	7	6	7
3	5	6	5	7	4	6	5	6.5	5	6
Median self‐reported sleepiness										
1	7	4	7	3	7	4	7	4	7	4
2	7	3	7	3	7	3	7	3	7	3
3	8	4	8	3	8	4	8	4	8	4

*Note*: Cognitive throughput (number of correct calculations), episodic memory (% words recalled correctly), working memory (spatial accuracy %), executive processing (reaction time), simple attention (reaction time).

## 8 Discussion

Previous work on this dataset has shown that sleep deprivation leads to impaired performance on all the cognitive tests except executive processing (Holding et al. 2021). The present study shows that the sleep‐deprived group had a slightly lower median performance compared to the well‐rested group on all cognitive tests except working memory. The sleep‐deprived group also had lower retrospective judgements of their performance compared to the well‐rested group. Interestingly, when estimating accuracy in terms of whether individuals who performed above the median also judged their performance as above the median, both groups were generally just above chance levels. In other words, participants who performed above the median were about equally likely to make a higher‐than‐median judgement as a judgement lower than the median, and vice versa. This was true for all five cognitive tests. While the sleep‐deprived individuals were numerically less accurate than the control group, this difference was small. It is possible that tasks or measures that see larger differences in performance are also more sensitive to the effects of sleep loss on metacognitive ratings.

Similar results were found for the relationship between sleepiness and metacognition. In other words, independent of the type of cognitive test, the accuracy of retrospective judgements when alert or when sleepy remained low. Individual global judgements for either group did not accurately reflect relative performance, that is, performance compared to other individuals.

The findings are in line with some of the earlier work, indicating that metacognitive accuracy remains fairly stable across sleep deprivation (Baranski 2007). Although the results of previous studies have been conflicting (for review, see Aidman et al. 2019; Boardman et al. 2021), including differences across different tests (e.g., Boardman et al. 2018), we show this pattern of results in a large sample, over five different cognitive tests, each with the same general pattern of preserved global confidence. In general, the judgements made were not much better than chance and the difference in accuracy between rested and sleep deprived was small if not negligible. Previous work has also suggested that at the individual level, metacognitive judgements are poor predictors of actual cognitive impairments due to sleep loss (Sallinen et al. 2013).

A limitation here is that our measure of accuracy is low in detail, especially given the bluntness of the analysis strategy. Although a strength of the current way of analysing is that we could combine performance and accuracy on all the tests without having to recode performance outcomes, future research would benefit from generating more detailed data, in which the metacognitive measure is closely connected to the performance. It would also be useful to have information on a test‐based item‐by‐item level. It may be that if we asked participants to judge performance on an item‐by‐item basis, the lowered fluency for sleep‐deprived participants would have lowered item‐by‐item confidence. This would allow for more detailed techniques to capture differences in both absolute and relative accuracy (Dunlosky and Metcalfe 2009), depending on sleep loss. In addition, although the sample was rather large, the between‐subjects setup is a limitation. A within‐subjects design would be optimal to determine individual variation in metacognitive accuracy after sleep loss.

Moreover, the data in this study do indicate that metacognitive ratings of sleep‐deprived or sleepy individuals' are lower than well‐rested individuals. Because performance on the test was also lower for the sleep‐deprived individuals, these lower judgements are, in a sense, correct, at least on a group level. However, the lower judgements did not correspond to accurately judging one's performance relative to others' performance. It could be that the retrospective performance judgements in the study were not based on a comparison between performance and a personal theory or norm of other individuals' performance (as assumed by the way that the data were analysed). Instead, it is possible that our participants used their experience of fluency during the tasks as an indicator of performance. That is, instead of comparing themselves to a performance norm, they may have used their level of processing fluency as a cue for performance (e.g., Schwartz and Jemstedt 2021), which may have been lower for the sleep‐deprived participants than the rested ones. This could explain the lower performance rating we observed in the sleep‐deprived group. At the same time, maybe fluency is not a cue that is sensitive enough to allow individuals to accurately judge themselves correctly above or below the median in performance. Furthermore, it may also be that what we see in our results is based on metacognitive beliefs. Prior research has shown that individuals use their metacognitive knowledge when making metacognitive judgements (Mueller and Dunlosky 2017), suggesting that the sleep‐deprived group should have lower performance judgements if they expect to be affected by sleep loss. The participants in our study may have believed that performance decreases in general for people who are sleep‐deprived, but perhaps this belief was not fine‐tuned enough to allow them to correctly rate themselves in relation to the median performance. Our future work aims to examine these different perspectives. Finally, it is possible that the lack of differences in metacognitive accuracy between the groups resulted from a floor effect in accuracy. Because the metacognitive accuracy was near chance level in both groups, it may have prevented any of the groups from being less accurate than the other. In other words, something in the judgement situation prevented participants from making accurate performance judgements, hindering either group from doing worse than the other. Such a floor effect may hide any differences in metacognitive accuracy between groups. As such, to avoid this possible problem, further research should focus on using tests and metacognitive measurements that allow participants higher levels of accuracy, to allow easier detection of differences.

As the focus here was on metacognitive judgements rather than behavioural adjustments, the question remains whether or not behaviour is changed because of expected performance (metacognitive control; Nelson and Narens 1990). This is an important question, as the answer would indicate whether sleep‐deprived individuals can be trusted to take appropriate action when performing a task on which they may be impaired, such as driving a truck, studying for an exam, or preparing for the operating room. In addition, the present study was conducted in a laboratory setting where the focus was on tests, and participants were prompted to judge their performance. The situation was likely unfamiliar to most participants, and most of the tasks, perhaps excluding the cognitive throughput test, were likely novel to them. Consequently, it is unclear whether the same pattern of results would be found in a less artificial context. Perhaps a sleepy driver, student, or surgeon can accurately monitor their cognitive performance when engaged in activities with which they have more experience in the task and how they perform the task when alert and sleepy. For sleepy driving, it is clear that people being aware of their sleepiness and impaired driving ability is predictive of actual driving impairment (e.g., Cai et al. 2024), but less is known about the extent to which they take action in these situations. There may also be a circadian effect in these kinds of ratings. Nishimura et al. (2023) found that nurses on a nightshift were more pessimistic about their performance on a simple reaction time test, even though there were no major impairments in actual performance. This finding still held when controlling for sleep duration and hours awake.

The present results were not designed to measure, nor do they shed light on, the mechanisms behind the retrospective performance judgements, other than sleepiness. That is, our data here do not tell us why the metacognitive judgements were lowered in the sleep‐deprived group; is it based on internal cues such as processing fluency or explicit metacognitive knowledge (Pintrich 2002) that being sleep‐deprived lowers performance in general? Nor do we know whether the judgement cues were affected by sleepiness. Although it is reasonable to believe that sleepiness affects the feeling of effort needed to perform a cognitive task (e.g., Engle‐Friedman 2014; Massar et al. 2019), there were no clear differences in the accuracy of metacognitive judgements based on differences in sleepiness as analysed in this study. Understanding more about the underlying mechanisms that influence sleep‐deprived individuals to accurately lower their judgements would be useful in developing aids to combat any inaccuracies in judgements and make predictions about situations in which metacognitive judgements may be more or less accurate due to sleep loss. For example, if the judgements are based on beliefs, then informing people about the hazards of sleep loss could help them to lower their judgements accurately. Additionally, if global judgements are mainly based on participants keeping track of their performance, we would expect them to be less accurate if the task they are doing is harder to keep track of, or if it does not have their full attention (e.g., driving a truck). Moreover, if judgements are made item‐by‐item, tracking‐performance effects may not override factors such as processing fluency. Importantly, the findings of the current study indicate that global metacognitive judgements such as these may not be appropriate to use in operational settings, as the accuracy in general was low.

## 9 Conclusion

In sum, the present study supports the conclusion that although people lower their retrospective performance judgements on tasks where their performance is lower because of sleep loss, there were no major effects on metacognitive accuracy. Indeed, metacognitive accuracy was generally low across sleep conditions. This result was consistent over five different cognitive tests, from simple reaction‐time tests to more demanding math problems and working memory tests. We encourage further research to elucidate the mechanisms behind metacognitive judgements made when sleepy and how they are influenced by sleep loss.

## Author Contributions

**Tina Sundelin:** conceptualization, investigation, funding acquisition, writing – original draft, methodology, validation, writing – review and editing, project administration, supervision, data curation. **Andreas Jemstedt:** conceptualization, investigation, funding acquisition, writing – original draft, methodology, validation, writing – review and editing, project administration. **Alvin Gavel:** visualization, formal analysis, data curation, writing – review and editing, methodology, writing – original draft. **Bennett L. Schwartz:** writing – review and editing, supervision, funding acquisition, investigation. **John Axelsson:** investigation, funding acquisition, writing – review and editing, supervision, resources.

## Conflicts of Interest

The authors declare no conflicts of interest.

## Supporting information


**Appendix S1.** Supporting information.

## Data Availability

The full results and analysis code can be found on https://github.com/AlvinGavel/Too_Tired_to_Know.
