# Supplementary material for: The Effect of Sleep Loss on Retrospective Metacognitive Judgements Across Five Cognitive Tests
Source: J Sleep Res. 2025 Aug 14;35(2):e70141. doi: 10.1111/jsr.70141 (PMC13003297; doi:10.1111/jsr.70141)
Supplement: Supplementary file 1 — Appendix S1. Supporting information. [file JSR-35-e70141-s001.docx]

Mathematical supplement

# Mathematical details

Our analysis is slightly unconventional, but in ways that we believe we can justify. A common way to do statistical analysis of hypotheses and data similar to ours would be to attempt to falsify the *null hypothesis* that the size of the effect under investigation is exactly zero and that systematic errors are exactly zero. In our case, the null hypothesis would be that the participants’ capacity for metacognition is identical between the test and control ground. Testing this null hypothesis would not be scientifically meaningful, since capacity for metacognition is a continuous quantity, meaning that it is *a priori* given that they will differ to some extent. Hence, it is guaranteed that given enough data our results would become ’statistically significant’, even if the actual effect was too small to be of any practical interest. This observation is unfortunately not limited to our study: There is a widespread tendency in the social sciences to ’falsify’ null hypotheses that are known *a priori* to be false. For a historical look at how long the issue has been discussed by statisticians, we refer the reader to [1]. In our analysis we follow [2] in defining a *practical significance level*, which essentially states the smallest effect size that we think is of any scientific interest.

In addition, our analysis is intended to respect the fact that our data exist on an ordinal scale, not an interval scale or higher level of measurement. That is, we can only say that one participant’s self-reported performance or sleepiness is higher than, lower than, or equal to another participant’s. There are no meaningful statements that can be made about *how much* they differ. The observed performances sometimes exists on an interval scale – for example, reaction speed – we cannot use this since we are attempting to investigate how they depend on the ordinal data. This means that concepts that are often used in statistical analysis, such as ‘linear relationship’ or ‘normal distribution’, cannot be used by us since they are simply not well defined for our kind of data.

Finally, our analysis uses a Bayesian framework rather than the more common frequentist. The reasons why we have chosen a this are partly philosophical but mostly pedagogical: There is a strong tendency among both readers of statistical analyses and statisticians themselves to interpret both frequentist *p*-values and Bayesian posteriors as though they were Bayesian posteriors. See [7] for a discussion of the problem, [3] for an empirical study of the tendency to misinterpret p-values, and [4] for an example of a statistics textbook that makes this mistake. Since we are not aware of any method to effectively prevent this pervasive misreading, we have decided that if the numbers we provide will be interpreted as Bayesian posteriors by most readers, it is better if they actually are Bayesian posteriors.

## Definitions and equations

We use *x* to denote either one of the test and control group, spelling out ‘test’ and ’cont.’ where we need to. For each group *x* there is some probability *P^x^* that a participant will rate themselves correctly with respect to the median. We denote the number of correct ratings by $n_{c}^{x}$ and the number of incorrect ratings by $n_{i}^{x}$.By Bayes’ theorem, the probability density for a given value of $P^{x}$, given the observed $n_{c}^{x}$ and $n_{i}^{x}$, is:

$p\left( P^{x} | n_{c}^{x},n_{i}^{x} \right)=\frac{p\left( n_{c}^{x},n_{i}^{x} | P^{x} \right)p\left( P^{x} \right)}{P\left( n_{c}^{x},n_{i}^{x} \right)}$ ($SEQ Equation \backslash* ARABIC$ $1$)

where $p\left( n_{c}^{x},n_{i}^{x} | P^{x} \right)$ is the likelihood for the observations, $p\left( P^{x} \right)$ is our prior and $P\left( n_{c}^{x},n_{i}^{x} \right)$ is effectively a normalization factor. For simplicity, we assume a flat

prior in the main body of the article, but discuss this choice more in Sect. 2.1.

The details of fitting binomial functions can be found in a standard textbook. Given the likelihood of $n_{c}^{x}$ correct ratings and $n_{i}^{x}$ incorrect ratings, given a particular value of $P^{x}$ is:

$p\left( P^{x} | n_{c}^{x},n_{i}^{x} \right)=\frac{\left( P^{x} \right)^{n_{c}^{x}}\left( 1-P^{x} \right)^{n_{i}^{x}}}{B\left( n_{c}^{x}+1,n_{i}^{x}+1 \right)} ($ $SEQ Equation \backslash* ARABIC$ $2$)

where $B$ is defined as:

$B\left( \alpha,\beta\right)=\frac{\Gamma\left( \alpha\right)\Gamma\left( \beta\right)}{\Gamma\left( \alpha+\beta\right)}$ ($SEQ Equation \backslash* ARABIC$ $3$)

where $\Gamma$ is the gamma function.

Strictly speaking (2) is not defined for $P^{x}=0$ when $n_{c}^{x}=0$ or for $P^{x}=1$ when $n_{i}^{x}=0$, since in both cases the numerator contains a factor of the form $0^{0}$. This is unlikely to matter in any realistic scenario, but it will occur later in this text where we test the robustness of the analysis by exploring some edge cases. To deal with that, we continuously extend the function by:


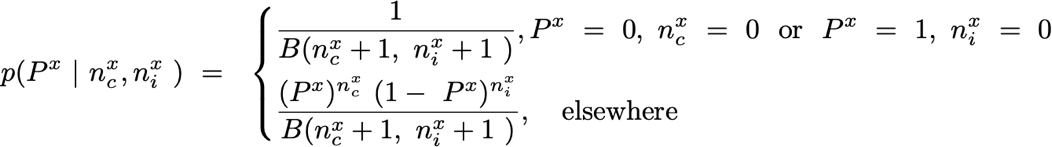
(4)

The probability distribution $p\left( D \right)$ over the difference $D\equiv P^{\text{test}}-P^{\text{cont.}}$ between the two groups is given by the convolution between the two probability distributions. The probability $P_{\text{prac}}$ that this difference lies in a particular interval is given by:

$P_{\text{prac}}=\int_{-S}^{S} p\left( D \right)dD$ (5)

Where $S$ is the practical significance level, which we have chosen to be 0*.*1. We discuss the impact of this choice in Sect. 2.2.

# Robustness checks

There are two steps in our statistical analysis that are conspicuously arbitrary: Our choice of prior and our definition of practically significant difference. In addition, there is our choice to split the participants based on whether they were intentionally deprived of sleep, rather than whether they reported actually feeling sleepy during the test, as well as our choice to calculate medians over both the test and control groups rather than over each group individually. In this section of the supplement we show the results that we could have gotten if we had made different choices in the analysis, in the hope of reassuring the reader that our conclusions are not just an artefact of those choices.

## Choice of prior

We have chosen a flat prior on $P^{\text{test}}$ and $P^{\text{cont.}}$. There is a widespread misconception in Bayesian statistics that a flat prior is the same thing as an uninformative prior, so to prevent this misreading we want to emphasise that our choice was made purely for convenience. In general, what prior counts as ‘flat’ depends on the parametrisation of the situation. That is, two physically equivalent models of a given situation, which only differ in formal details such as the choice of coordinates to use, can have flat priors that are not themselves physically equivalent. See [6] for a deeper discussion of this.

In our case, the choice of a flat prior on $P^{\text{test}}$ and $P^{\text{cont.}}$ is equivalent to assuming a peaked prior on D, shown in Fig. 1. This means that we start out with a mild preference for the hypothesis that $P^{\text{test}}$ and $P^{\text{cont.}}$ are similar. Given the choice of $S = 0.1$, this corresponds to a prior probability of 81% that there is a practically significant difference. Given that this probability ends up being below one percent after the analysis, we can safely say that our conclusions are not predetermined by the choice of prior.


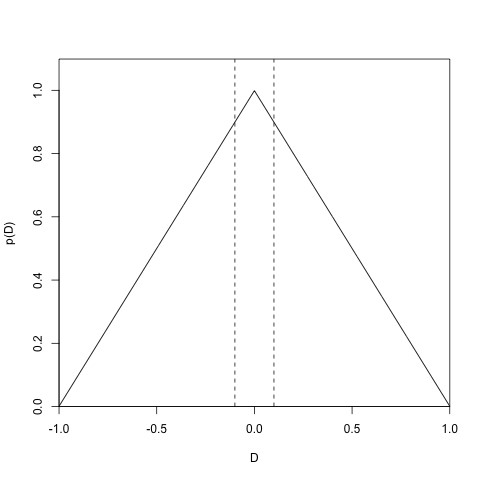


Figure 1: *The prior probability density on the difference* $D$*. This can be given by inserting* $n_{c}^{\text{cont.}}=n_{i}^{\text{cont.}}=n_{c}^{\text{test}}=n_{i}^{\text{test}}=0$ *in* (4)*.*

## The practical significance level

There is no strong motivation behind our choice of $S = 0.1$. Hence, we look at what conclusions we might have drawn from our data if we had chosen, for example, 0*.*01, 0*.*05, or 0*.*2^^[[1]](#footnote-1)^^. Table 1 shows the probabilities of practically significant results for some different choices of $S$. In each case we split the participants according to the pre-assigned groups, not self-reported sleepiness.

| $S$ | $P\left( \left\vert D \right\vert>S \right)$ |
| --- | --- |
| 1% | 92% |
| 5% | 28% |
| 10% | *>* 1% |
| 20% | *>* 1% |

Table 1: *Probability of practically significant difference* $D$ *for some choices of the significance level* $S$*.*

The choice also interacts with the prior in an important way, since some choices of *S* constrain the conclusions so strongly that they would say more about the choice of prior than about the data itself. To investigate this, we will also look at the conclusions for two limiting cases of what the data could have looked like: First, we look at the most unambiguous data possible, with all participants in the control group categorising themselves correctly and no participants in the test group doing so. Second, we will look at the case of no data at all, where the conclusion is formed entirely by the prior.

The $P\left( \left| D \right|>S \right)$ for these edge cases, for different values of $S$, are shown in Table 2. In the case of unambiguous data the conclusion of there being an effect is so close to certain as to make no difference for every choice of *S*. In the case of no data we can see that the prior is weakly in favour of there being an effect for values around our preferred choice of *S* = 0*.*1.

| $S$ | $P\left( \left\vert D \right\vert>S \right)$ | |
| --- | --- | --- |
|  | Unambiguous data | No data |
| 1% | ≈ 100% | 98% |
| 5% | ≈ 100% | 90% |
| 10% | ≈ 100% | 81% |
| 20% | ≈ 100% | 64% |

Table 2: *Probability of practically significant difference* $D$ *in the edge case of the data being as unambiguous as they can be, and there being no data whatsoever, for the same choices of the significance level S as in Table. 1.*

## Division of participants

During the planning stage of the statistical analysis we considered splitting participants by their reported sleepiness. This might have allowed us to disentangle the effects of the physiological state of being sleep-deprived, and the subjective experience of sleepiness. We refer the reader to [5] for a detailed discussion of causal graphs, but Fig. 2 describes the causal model that we were considering.

Sleep-deprivation

Physiological effects of

sleep deprivation

Subjective feeling of

sleepiness

Actual performance

Estimated performance

Figure 2: *DAG describing the causal model that could have gone into an analysis to disentangle the effects of the physiological state of sleep deprivation and the subjective experience of sleepiness.*

In the end we decided not to pursue this path of investigation, but we still tested the robustness of the analysis by – somewhat arbitrarily – dividing the participants into a ‘test group’ with above-median reported sleepiness and a ‘control group’ with below-median reported sleepiness. For the different choices of *S*, this gives the probabilities of a practically significant difference shown in Table 3. This shows that if the choice had been made to split by reported sleepiness, this would have led to stronger support for the conclusion that there is no practically significant difference.

| $S$ |  | $P\left( \left\vert D \right\vert>S \right)$ |
| --- | --- | --- |
|  | Pre-set split | Split by reported sleepiness |
| 1% | *89.*18% | 67*.*92% |
| 5% | 21*.*10% | 3*.*26% |
| 10% | 0*.*06% | ≈ 0*.*00% |
| 20% | ≈ 0*.*00% | ≈ 0*.*00% |

Table 3: *Probability of practically significant difference* $D$ *for two different ways of separating the test and control group, for the same choices of the significance level* $S$ *as in Table. 1.*

## Choice of median

When we compare the participants’ actual and estimated performance, we compare them to the median across both the control and test group. This implicitly assumes that the participants are trying to rate themselves on an absolute scale, so that if a sleep-deprived participant performs worse than they would have done after a full night’s sleep, but rates themselves the same as they would have then, then that participant shows poor metacognition. This assumption may be incorrect. The participant could be fully aware that they are performing worse, but attempting to rate themselves with respect to how well they expect sleep-deprived people to perform. In the latter case, the median we should compare them to would be the median over the test group.

We have no way of finding out, after the fact, what the participants implicitly assumed that they should be rating themselves with respect to. Most likely, most people are not trying to rate themselves according to anything as well-defined as the two scales we describe above. However, we can assume that what they are trying to do is somewhere in between those two scales. Hence, we can test the robustness of our conclusions by seeing if they change noticeably if we calculate the probability of practically significant difference using the within-groups medians.

We compare the results for our preferred analysis to that using the within-groups median in Table. 4. Our conclusions would be somewhat weaker if within-groups medians were used – as one would expect, given that this essentially adds another free parameter to the analysis.

| $S$ | $P\left( \left\vert D \right\vert>S \right)$ |
| --- | --- |
|  | Across-groups median Within-groups median |
| 1% | 89*.*18% 94*.*35% |
| 5% | 21*.*10% 33*.*67% |
| 10% | 0*.*06% 0*.*20% |
| 20% | ≈ 0*.*00% ≈ 0*.*00% |

Table 4: *Probability of practically significant difference* $D$ *for two different choices of set to calculate medians over, for the same choices of the significance level*$S$ *as in Table. 1.*

# References

1. J. Cohen. The earth is round (p < .05). *American Psychologist*,

49(12):997–1003, 1994.

1. W. M. Goodman, S. E. Spruill, and E. Komaroff. A proposed hybrid effect size plus *p*-value criterion: Empirical evidence supporting its use. *The American Statistician*, 73(sup1):168–185, Mars 2019.
2. R. Hubbard and M. J. Bayarri. Confusion over measures of evidence (*p*’s) versus errors (*α*’s) in classical statistical testing. *The American Statistician*, 57(3):171–178, Augusti 2003.
3. H. Michael and T. Gordon. *Medical Statistics Made Easy*. Scion Publishing Ltd, 3 edition, 2014.
4. J. Pearl and D. Mackenzie. *The Book of Why*. Penguin, 2018.
5. U. von Toussaint. Bayesian inference in physics. *Reviews of Modern Physics*, 83:943–999, July-September 2011.
6. R. L. Wasserstein and N. A. Lazar. The ASA statement on *p*-values: Context, process, and purpose. *The American Statistician*, 70(2):129–133, Maj 2016.

1. If we set $S = 0$ then Eq. (5) means that we immediately get $P_{\text{prac}}=1$, reflecting our observation in the introduction that the null hypothesis of no effect is necessarily false. [↑](#footnote-ref-1)
